# Effects of surgery on survival of patients aged 75 years or older with Merkel cell carcinoma

**DOI:** 10.1002/cam4.4437

**Published:** 2021-11-24

**Authors:** Kehui Ren, Xufeng Yin, Bingrong Zhou

**Affiliations:** ^1^ Department of Dermatology The First Affiliated Hospital of Nanjing Medical University Nanjing China

**Keywords:** elderly, Merkel cell carcinoma, prognosis, surgery

## Abstract

**Objective:**

To investigate whether surgery improves prognosis in elderly patients with Merkel cell carcinoma (MCC).

**Materials/Methods:**

Data of all patients with MCC diagnosed between 2004 and 2015 were extracted from the Surveillance, Epidemiology, and End Results (SEER) database. Differences in baseline characteristics were analyzed among the age groups (75–80, 80–85, and ≥85 years). Multivariate Cox proportional hazards analysis was used to assess the effects of each variable on patient outcomes. The Kaplan–Meier curves were employed to evaluate MCC overall survival (OS) and MCC‐specific survival (MSS).

**Results:**

A total of 1156 of patients with MCC met the inclusion and exclusion criteria. The surgery rate decreased with age (75–80, 80–85, and ≥85 years were 93.3%, 91.1%, and 88.7%, respectively; *p* = 0.082). Multivariate Cox proportional hazards analysis showed that the OS of patients in the 80–85 years group (hazard ratio [HR] = 1.39; 95% confidence interval [CI] = 1.14–1.70; *p* = 0.001) and the ≥85 years group (HR = 2.18; 95% CI = 1.80–2.63; *p* < 0.0001) was worse than that in the 75–80 years group. Compared with the non‐surgery groups, the HR for the surgery group was 0.75 for OS (95% CI = 0.56–1.00; *p* = 0.048) and 0.73 for MSS (95% CI = 0.48–1.10; *p* = 0.130). Subgroup analyses showed that patients aged ≥85 years undergoing surgery had better OS (HR = 0.65; 95% CI = 0.45–0.95; *p* = 0.024).

**Conclusions:**

MCC patients aged 75 years and older would benefit from surgical resection. However, surgical resection should be performed cautiously, and larger prospective clinical trials are needed to further verify these findings.

## Graphical abstract

We find surgery can improve the prognosis of elderly patients with Merkel cell carcinoma (MCC), especially for patients aged 85 years or older with MCC would benefit from surgical resection by doing subgroup analysis.

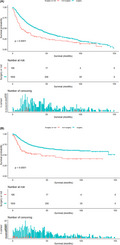

## 1 INTRODUCTION

Merkel cell carcinoma (MCC) is a relatively rare but highly aggressive cutaneous neuroendocrine carcinoma for which long‐term ultraviolet irradiation and Merkel cell polyomavirus infection are the main etiological factors.^1^ MCC lesions are characterized by rapidly growing, painless, red or purplish‐blue skin nodules that have a high incidence of local relapse and lymph node metastasis.^2^, ^3^ The prognosis for MCC is stage‐dependent, with a 51% 5‐year overall survival (OS) rate for localized cancers, and 35% and 14% for regional and distant metastases, respectively.^4^, ^5^, ^6^ In recent years, the incidence and mortality rate of MCC have increased significantly.^7^, ^8^

Clinical treatments for MCC include surgical resection, radiation therapy, chemotherapy, immunotherapy, etc. Surgery is widely used clinically, but its outcome may be influenced by the location and size of the lesions, postoperative sequelae, and contraindications to anesthesia.^9^ The typical surgical rate is not yet clear. However, according to National Comprehensive Cancer Network (NCCN) guidelines, the standard treatment for patients with primary MCC is surgical removal followed by adjuvant radiation therapy.^10^

The elderly, the most common population that develops this malignancy, experience a great deal of suffering with MCC, and population‐based studies show that MCC incidence increases among people older than 85 years old.^11^, ^12^, ^13^ The incidence of MCC in older population is expected to increase by up to 67% between 2010 and 2030.^14^ Studies have shown that a poor prognosis is related to tumor metastasis and various diverse treatments in elderly patients.^15^, ^16^, ^17^ However, older patients have been heavily under‐represented in clinical trials, and only 8.3% of the participants were over 75 years old.^18^

Access to real‐world clinical data enables a retrospective study of outcomes in older patients. Hopefully, the utilization of variables related to MCC in the Surveillance, Epidemiology, and End Results (SEER) database may assist clinicians in their decision‐making when confronted with elderly patients with MCC. Current research based on the SEER database investigated the prognostic factors of elderly patients with MCC, but no study to date has explored the prognosis of surgical patients older than 75 years.^19^, ^20^ Therefore, the aim of this study was to evaluate the OS and MCC‐specific survival (MSS) of patients over 75 years of age who received surgical resection versus those of patients who did not.

## 2 MATERIALS AND METHODS

### 2.1 Databases and patient selection

The demographic and clinicopathological features of patients with MCC diagnosed between 2004 and 2015 were extracted from the SEER database using SEER*Stat software version 8.3.9 (http://seer.cancer.gov/). Since the patients’ personal information was not included in the database, neither informed consent from patients nor approval from the ethics committee was required. Based on the purpose of this research, this study only included patients who were 75 years or older. All included patients were grouped according to age^21^: 75–80, 80–85, and ≥85 years. Each patient was assigned an International Classification of Diseases for Oncology (3rd Edition) according to MCC histologic type (8247/3) and primary site (44.0–44.9). Other clinicopathological parameters, including ethnicity, gender, marital status, tumor size, lymph node metastasis, primary site, tumor necrosis metastasis (TNM) staging, AJCC staging, surgery, radiotherapy, chemotherapy, and presence or absence of a distant metastasis, were included. Tumor size was divided into two groups (<2 and ≥2 cm). The primary tumor site was stratified into five groups: face, head/neck, trunk, limbs/shoulder/hip, and other. The exclusion criteria were as follows: (1) diagnosis of MCC not confirmed histopathologically; (2) unknown ethnicity; (3) unknown marital status; (4) unknown tumor size; (5) unknown lymph node or distant metastasis information; (6) unknown stage information; (7) unknown therapy information; and (8) two or more cancers. Treatments included surgery, radiotherapy, and chemotherapy. According to the difference in surgical excision margin (EM), treatment options were divided into six groups^19^: (1) no surgery; (2) local destruction (LD), including electrocautery, laser excision, and excisional biopsy; (3) EM ≤1 cm, including shave biopsy, punch biopsy, incisional biopsy followed by gross excisions, and Mohs surgery with 1‐cm margin or less; (4) 1 cm < EM ≤ 2 cm, including Mohs surgery with more than 1‐cm margin, wide excision, or re‐excision of lesion or minor (local) amputation with margins more than 1 cm, clear margins more than 1 cm, and ≤2 cm record; (5) EM >2 cm, including surgical margins >2 cm and major amputation; and (6) the specific mode of operation is unknown.

### 2.2 Data analysis

The statistical analyses were performed using Empowerstats software (X&Y Solutions) and statistical package R V.3.5.2 (http://www.r‐project.org). Normally distributed data are presented as mean ± standard deviation, while skewed data are shown as median (25th–75th percentiles) and categorical data are shown as number and percentage (%). The clinicopathologic categorical variables were compared using the Pearson chi‐squared test. We created a matching data set using propensity score matching (PSM) to avoid bias and balance covariates (age, gender, race, marriage, primary site, and AJCC stage) between surgical and nonsurgical groups. PSM was performed using a 1:1 nearest neighbor match to create a match between the surgical and nonsurgical groups. The indicators of prognostic assessment were OS and MSS. OS was defined as the time, in months, from diagnosis to death or last follow‐up. MSS was defined as the time, in months, from diagnosis to death caused by MCC or last follow‐up. The multivariate analysis based on the Cox proportional hazards model was performed to evaluate independent prognostic factors of elderly patients with MCC, and the effects are expressed as hazard ratio (HR) and 95% confidence interval (CI). The prognostic effect of surgery on the clinical outcomes of the elderly patients was evaluated by the Kaplan–Meier method, while survival curves were estimated using the log‐rank test. The patients were divided into subgroups based on different clinicopathological features and subgroup analysis was performed to determine the effect of surgery on clinical prognosis. *p* < 0.05 were considered statistically significant.

## 3 RESULTS

### 3.1 Basic patient information

A total of 1156 patients with MCC were finally included in this population‐based study. Among them, 334 (29.8%) were in the 75–80 years group, 360 (31.1%) were in the 80–85 years group, and 452 (39.1%) were in the ≥85 years group. The rate of surgery decreased with age (93.9%, 91.1%, and 88.7% in the 75–80, 80–85, and ≥85 years groups, respectively). Chemotherapy rates were 9.9%, 10.0%, and 3.1% for the 75–80, 80–85, and ≥85 years groups, respectively. The OS rate of MCC decreased with age (48.0%, 37.5%, and 22.1% in the 75–80, 80–85, and ≥85 years groups, respectively). Table 1 lists the patients’ baseline characteristics at the time of diagnosis by age group.

**TABLE 1:** Basic characteristics of elderly patients with Merkel cell carcinoma in different age groups

Age	75–80 years	80–85 years	≥85 years	*p* value
*N*	344	360	452	
Survival time (months), median (inter‐quartile range)	30.5 (14.0–59.0)	26.0 (12.0–50.0)	19.0 (9.0–37.0)	<0.001
Race, *n* (%)				0.109
White	333 (96.8%)	343 (95.3%)	425 (94.0%)	
Black	1 (0.3%)	8 (2.2%)	13 (2.9%)	
Other	10 (2.9%)	9 (2.5%)	14 (3.1%)	
Sex, *n* (%)				0.069
Female	141 (41.0%)	160 (44.4%)	222 (49.1%)	
Male	203 (59.0%)	200 (55.6%)	230 (50.9%)	
Marriage, *n* (%)				<0.001
Married	207 (60.2%)	188 (52.2%)	178 (39.4%)	
Single	18 (5.2%)	21 (5.8%)	33 (7.3%)	
Separated/widowed/divorced/unmarried	97 (28.2%)	136 (37.8%)	201 (44.5%)	
Unknown	22 (6.4%)	15 (4.2%)	40 (8.8%)	
Tumor size, *n* (%)				0.41
<2 cm	199 (57.8%)	199 (55.3%)	240 (53.1%)	
≥2 cm	145 (42.2%)	161 (44.7%)	212 (46.9%)	
Lymph nodes, *n* (%)				0.255
No lymph involved	231 (67.2%)	257 (71.4%)	327 (72.3%)	
Lymph involved	113 (32.8%)	103 (28.6%)	125 (27.7%)	
Primary site, *n* (%)				0.014
Face	127 (36.9%)	133 (36.9%)	179 (39.6%)	
Head/neck	29 (8.4%)	33 (9.2%)	70 (15.5%)	
Trunk	25 (7.3%)	31 (8.6%)	38 (8.4%)	
Limbs/shoulder/hip	150 (43.6%)	151 (41.9%)	156 (34.5%)	
Other	13 (3.8%)	12 (3.3%)	9 (2.0%)	
T stage, *n* (%)				0.784
T0	12 (3.5%)	11 (3.1%)	9 (2.0%)	
T1	201 (58.4%)	208 (57.8%)	253 (56.0%)	
T2	97 (28.2%)	96 (26.7%)	134 (29.6%)	
T3	16 (4.7%)	24 (6.7%)	32 (7.1%)	
T4	18 (5.2%)	21 (5.8%)	24 (5.3%)	
N stage, *n* (%)				0.255
N0	231 (67.2%)	257 (71.4%)	327 (72.3%)	
N1	113 (32.8%)	103 (28.6%)	125 (27.7%)	
M stage, *n* (%)				0.917
M0	321 (93.3%)	337 (93.6%)	425 (94.0%)	
M1	23 (6.7%)	23 (6.4%)	27 (6.0%)	
AJCC stage, *n* (%)				0.52
I	149 (43.3%)	172 (47.8%)	208 (46.0%)	
II	63 (18.3%)	69 (19.2%)	100 (22.1%)	
III	109 (31.7%)	96 (26.7%)	117 (25.9%)	
IV	23 (6.7%)	23 (6.4%)	27 (6.0%)	
Surgery, *n* (%)				0.082
No	23 (6.7%)	32 (8.9%)	51 (11.3%)	
Yes	321 (93.3%)	328 (91.1%)	401 (88.7%)	
Surgical procedure, *n* (%)				<0.001
No surgery	23 (6.7%)	32 (8.9%)	51 (11.3%)	
LD	52 (15.1%)	71 (19.7%)	132 (29.2%)	
EM ≤1 cm	112 (32.6%)	93 (25.8%)	123 (27.2%)	
1 cm < EM ≤ 2 cm	112 (32.6%)	129 (35.8%)	111 (24.6%)	
EM >2 cm	27 (7.8%)	23 (6.4%)	11 (2.4%)	
Unknown	18 (5.2%)	12 (3.3%)	24 (5.3%)	
Radiation, *n* (%)				<0.001
No/unknown	145 (42.2%)	171 (47.5%)	264 (58.4%)	
Yes	199 (57.8%)	189 (52.5%)	188 (41.6%)	
Chemotherapy, *n* (%)				<0.001
No/unknown	310 (90.1%)	324 (90.0%)	438 (96.9%)	
Yes	34 (9.9%)	36 (10.0%)	14 (3.1%)	
Distant metastases, *n* (%)				0.917
No	321 (93.3%)	337 (93.6%)	425 (94.0%)	
Yes	23 (6.7%)	23 (6.4%)	27 (6.0%)	
MCC‐specific survival				0.856
Alive/dead of other cause	239 (69.5%)	257 (71.4%)	318 (70.4%)	
Dead	105 (30.5%)	103 (28.6%)	134 (29.6%)	
Overall survival				<0.001
Alive	165 (48.0%)	135 (37.5%)	100 (22.1%)	
Dead	179 (52.0%)	225 (62.5%)	352 (77.9%)	

Note

Results in the table: median (Q1–Q3)/*N* (%). *p* value: the Kruskal–Wallis rank sum test is used for continuous variables. If the count variable has a theoretical number <10, the Fisher's exact probability test is used.

Abbreviations: EM, excision margin; LD, local destruction; MCC, MCC‐specific survival.

Table 2 lists clinical characteristics before and after PSM in the surgical and nonsurgical groups. Compared with the nonsurgical group, the surgery group had fewer lymph node metastasis cases (*n* = 762; 72.6%), more AJCC stage I cases (*n* = 509; 48.5%), and more lesions located on the face (*n* = 410; 39.0%) and limbs/shoulder/hip (*n* = 434; 41.3%).

**TABLE 2:** Basic characteristics of elderly patients with Merkel cell carcinoma by surgery groups

Surgery	Before matching			After matching		
	No surgery	Surgery	*p* value	No surgery	Surgery	*p* value
*N*	106	1050		106	106	
Survival time (months), median (inter‐quartile range)	12.5 (6.0–30.2)	25.0 (12.0–48.0)	0.002	12.5 (6.0–30.2)	19.0 (7.2–34.8)	0.508
Age, *n* (%)			0.082			0.432
75–80	23 (21.7%)	321 (30.6%)		23 (21.7%)	21 (19.8%)	
80–85	32 (30.2%)	328 (31.2%)		32 (30.2%)	25 (23.6%)	
≥85	51 (48.1%)	401 (38.2%)		51 (48.1%)	60 (56.6%)	
Race, *n* (%)			0.633			0.135
White	99 (93.4%)	1002 (95.4%)		99 (93.4%)	90 (84.9%)	
Black	3 (2.8%)	19 (1.8%)		3 (2.8%)	6 (5.7%)	
Other	4 (3.8%)	29 (2.8%)		4 (3.8%)	10 (9.4%)	
Sex, *n* (%)			0.533			0.169
Female	51 (48.1%)	472 (45.0%)		51 (48.1%)	61 (57.5%)	
Male	55 (51.9%)	578 (55.0%)		55 (51.9%)	45 (42.5%)	
Marital status, *n* (%)			0.052			0.374
Married	43 (40.6%)	530 (50.5%)		43 (40.6%)	35 (33.0%)	
Single	7 (6.6%)	65 (6.2%)		7 (6.6%)	11 (10.4%)	
Separated/widowed/divorced/unmarried	43 (40.6%)	391 (37.2%)		43 (40.6%)	51 (48.1%)	
Unknown	13 (12.3%)	64 (6.1%)		13 (12.3%)	9 (8.5%)	
Tumor size, *n* (%)			0.608			<0.001
<2 cm	56 (52.8%)	582 (55.4%)		56 (52.8%)	31 (29.2%)	
≥2 cm	50 (47.2%)	468 (44.6%)		50 (47.2%)	75 (70.8%)	
Lymph nodes, *n* (%)			<0.001			0.008
No lymph involved	53 (50.0%)	762 (72.6%)		53 (50.0%)	34 (32.1%)	
Lymph involved	53 (50.0%)	288 (27.4%)		53 (50.0%)	72 (67.9%)	
Primary site, *n* (%)			<0.001			<0.001
Face	30 (28.3%)	410 (39.0%)		30 (28.3%)	16 (15.1%)	
Head/neck	19 (17.9%)	112 (10.7%)		19 (17.9%)	6 (5.7%)	
Trunk	3 (2.8%)	91 (8.7%)		3 (2.8%)	14 (13.2%)	
Limbs/shoulder/hip	25 (23.6%)	434 (41.3%)		25 (23.6%)	69 (65.1%)	
Other	29 (27.4%)	3 (0.3%)		29 (27.4%)	1 (0.9%)	
T stage, *n* (%)			<0.001			<0.001
T0	30 (28.3%)	2 (0.2%)		30 (28.3%)	1 (0.9%)	
T1	28 (26.4%)	634 (60.4%)		28 (26.4%)	31 (29.2%)	
T2	28 (26.4%)	299 (28.5%)		28 (26.4%)	48 (45.3%)	
T3	13 (12.3%)	59 (5.6%)		13 (12.3%)	14 (13.2%)	
T4	7 (6.6%)	56 (5.3%)		7 (6.6%)	12 (11.3%)	
N stage, *n* (%)			<0.001			0.008
N0	53 (50.0%)	762 (72.6%)		53 (50.0%)	34 (32.1%)	
N1	53 (50.0%)	288 (27.4%)		53 (50.0%)	72 (67.9%)	
M stage, *n* (%)			<0.001			0.871
M0	81 (76.4%)	1002 (95.4%)		81 (76.4%)	82 (77.4%)	
M1	25 (23.6%)	48 (4.6%)		25 (23.6%)	24 (22.6%)	
AJCC stage, *n* (%)			<0.001			<0.001
I	20 (18.9%)	509 (48.5%)		20 (18.9%)	3 (2.8%)	
II	24 (22.6%)	208 (19.8%)		24 (22.6%)	16 (15.1%)	
III	37 (34.9%)	285 (27.1%)		37 (34.9%)	63 (59.4%)	
IV	25 (23.6%)	48 (4.6%)		25 (23.6%)	24 (22.6%)	
Radiation, *n* (%)			<0.001			0.203
No/unknown	36 (34.0%)	544 (51.8%)		36 (34.0%)	45 (42.5%)	
Yes	70 (66.0%)	506 (48.2%)		70 (66.0%)	61 (57.5%)	
Chemotherapy, *n* (%)			<0.001			0.073
No/unknown	82 (77.4%)	990 (94.3%)		82 (77.4%)	92 (86.8%)	
Yes	24 (22.6%)	60 (5.7%)		24 (22.6%)	14 (13.2%)	
Distant metastases, *n* (%)			<0.001			0.871
No	81 (76.4%)	1002 (95.4%)		81 (76.4%)	82 (77.4%)	
Yes	25 (23.6%)	48 (4.6%)		25 (23.6%)	24 (22.6%)	
MCC‐specific survival			<0.001			0.89
Alive/dead of other cause	59 (55.7%)	755 (71.9%)		59 (55.7%)	60 (56.6%)	
Dead	47 (44.3%)	295 (28.1%)		47 (44.3%)	46 (43.4%)	
Overall survival			0.003			0.735
Alive	23 (21.7%)	377 (35.9%)		23 (21.7%)	21 (19.8%)	
Dead	83 (78.3%)	673 (64.1%)		83 (78.3%)	85 (80.2%)	

Abbreviation: MCC, MCC‐specific survival.

The patients’ baseline characteristics at the time of diagnosis between survival group and death group are listed in Table S1. The result showed that significant decrease in survival with the increase in age (41.2%, 33.8%, and 25.0% in the 75–80, 80–85, and ≥85 years groups, respectively). Non‐surgery rates were 5.8% and 11.0% for the survival group and death group, respectively.

### 3.2 Survival analyses

Before PSM, there were statistically significant differences in MSS (non‐surgery: 55.7%; surgery: 71.9%, *p* < 0.001) and OS (non‐surgery: 21.7%; surgery: 35.9%, *p* = 0.003) between the surgical and nonsurgical groups. But after PSM, the result showed no statistically significant differences in MSS (non‐surgery: 55.7%; surgery: 56.6%, *p* = 0.89) and OS (non‐surgery: 21.7%; surgery: 19.8%, *p* = 0.735) between the two groups.

**FIGURE 1:**
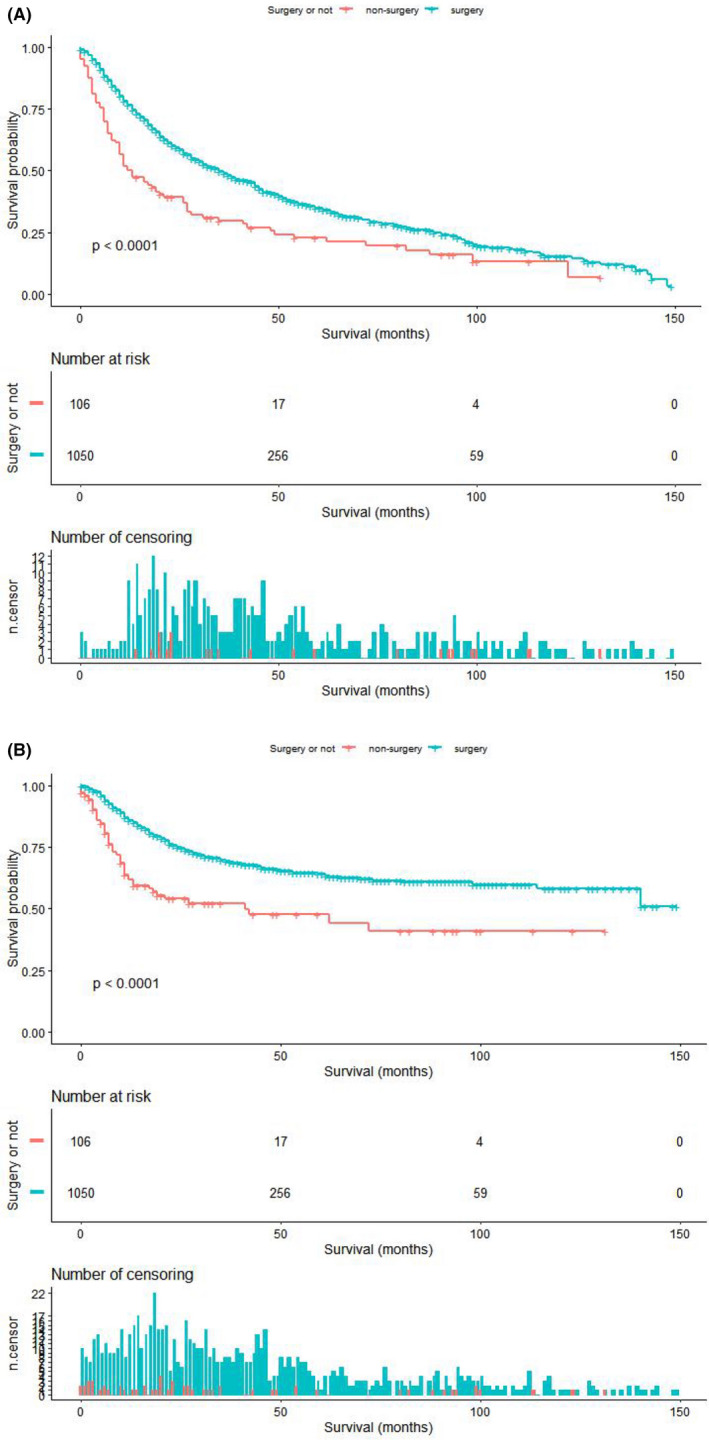
The Kaplan–Meier analysis of OS (A) and MSS (B) in the surgery and non‐surgery groups. MSS, MCC‐specific survival; OS, overall survival

Multivariate Cox proportional hazards analyses (Table 3) showed that age, ethnicity, gender, marital status, primary site, TNM stage, and surgery were independent prognostic factors for OS of MCC patients. The analysis showed that the OS of patients in the 80–85 years group (HR = 1.39; 95% CI = 1.14–1.70; *p* = 0.001) and ≥85 years old (HR = 2.18; 95% CI = 1.80–2.63; *p* < 0.0001) was worse than that of patients in the 75–80 years group. Relative to the non‐surgery groups, the HR for OS for the surgery groups was 0.75 (95% CI = 0.56–1.00; *p* = 0.048). As for MSS, sex, primary site, and TNM stage were the prognostic factors. MSS was not significantly different between the 80–85 years group (HR = 1.02, 95% CI = 0.77–1.35; *p* = 0.880) and the ≥85 years group (HR = 1.22; 95% CI = 0.93–1.59; *p* = 0.146) compared to the younger (75–80 years) patients. Compared with the non‐surgery group, the HR of MSS for the surgery groups was 0.73 (95% CI = 0.48–1.10; *p* = 0.130), showing no statistical significance. The Kaplan–Meier analysis (Figure 1) indicated that the surgery group had significantly better OS and MSS than the non‐surgery group. OS and MSS of different groups were analyzed by the Kaplan–Meier method, and the results are shown in Figures S1 and S2, respectively.

**TABLE 3:** Multivariate Cox proportional hazards analyses of elderly patients with Merkel cell carcinoma

Characteristics	Overall survival		MCC‐specific survival	
	HR (95% CI)	*p* value	HR (95% CI)	*p* value
Age				
75–80 years	1		1	
80–85 years	1.39 (1.14, 1.70)	0.001	1.02 (0.77, 1.35)	0.880
≥85 years	2.18 (1.80, 2.63)	<0.0001	1.22 (0.93, 1.59)	0.146
Race				
White	1		1	
Black	1.05 (0.60, 1.83)	0.866	1.37 (0.63, 2.98)	0.425
Other	0.49 (0.29, 0.84)	0.009	0.50 (0.24, 1.07)	0.074
Sex				
Female	1		1	
Male	1.39 (1.18, 1.63)	<0.0001	1.34 (1.05, 1.72)	0.019
Marital status				
Married	1		1	
Single	0.94 (0.68, 1.29)	0.684	1.14 (0.74, 1.75)	0.565
Separated/widowed/divorced/unmarried	1.20 (1.01, 1.43)	0.037	1.27 (0.99, 1.64)	0.063
Unknown	1.17 (0.87, 1.58)	0.298	0.96 (0.57, 1.63)	0.886
Primary site				
Face	1		1	
Head/neck	1.58 (1.24, 2.00)	0.000	1.85 (1.31, 2.62)	0.001
Trunk	1.08 (0.82, 1.44)	0.572	1.43 (0.98, 2.10)	0.065
Limbs/shoulder/hip	0.98 (0.82, 1.17)	0.816	0.87 (0.67, 1.14)	0.329
Other	4.30 (1.10, 16.81)	0.036	13.79 (2.37, 80.20)	0.004
T stage				
T0	1		1	
T1	4.63 (1.15, 18.67)	0.031	12.97 (2.03, 82.74)	0.007
T2	5.73 (1.42, 23.08)	0.014	14.72 (2.29, 94.39)	0.005
T3	6.03 (1.47, 24.71)	0.013	20.09 (3.07, 131.51)	0.002
T4	6.65 (1.61, 27.43)	0.009	17.89 (2.71, 118.23)	0.003
N stage				
N0	1		1	
N1	1.67 (1.41, 1.97)	<0.0001	2.64 (2.08, 3.34)	<0.0001
M stage				
M0	1		1	
M1	2.53 (1.94, 3.32)	<0.0001	4.11 (2.97, 5.67)	<0.0001
Surgery				
No	1		1	
Yes	0.75 (0.56, 1.00)	0.048	0.73 (0.48, 1.10)	0.130

Abbreviations: CI, confidence interval; HR, hazard ratio; MCC, MCC‐specific survival.

### 3.3 Subgroup analysis of clinical prognosis

To further explore the association between surgery and clinical prognosis, we performed a subgroup analysis of these clinicopathological variables (Table 4). Surgery was dramatically correlated with better OS in the ≥85 years group (HR = 0.65; 95% CI = 0.45–0.95; *p* = 0.024), while it was not significantly associated with MSS (HR = 0.73; 95% CI = 0.40–1.34; *p* = 0.309). The relationship between surgery and OS was not significant in the 75–80 years group (HR = 0.79; 95% CI = 0.34–1.82; *p* = 0.580) or the 80–85 years group (HR = 0.83; 95% CI = 0.46–1.47; *p* = 0.515).

**TABLE 4:** The effect of surgery on overall survival and cancer‐specific survival based on different subgroup variables

Characteristics	*N*	Overall survival		MCC‐specific survival	
		HR (95% CI)	*p* value	HR (95% CI)	*p* value
Age					
75–80 years	344	0.79 (0.34, 1.82)	0.580	0.52 (0.19, 1.44)	0.209
80–85 years	360	0.83 (0.46, 1.47)	0.515	0.66 (0.29, 1.48)	0.311
≥85 years	452	0.65 (0.45, 0.95)	0.024	0.73 (0.40, 1.34)	0.309
Sex					
Female	523	0.75 (0.49, 1.15)	0.194	0.64 (0.34, 1.20)	0.166
Male	633	0.74 (0.50, 1.10)	0.134	0.81 (0.47, 1.42)	0.465
Primary site					
Face	439	1.08 (0.65, 1.78)	0.773	0.75 (0.36, 1.58)	0.451
Head/neck	132	0.65 (0.33, 1.25)	0.197	0.84 (0.30, 2.33)	0.737
Trunk	94	0.42 (0.09, 2.02)	0.282	0.19 (0.03, 1.39)	0.103
Limbs/shoulder/hip	457	0.65 (0.39, 1.10)	0.112	0.57 (0.27, 1.19)	0.134
Other	34	1.02 (0.14, 7.49)	0.987	0.65 (0.05, 8.91)	0.749
T stage					
T0	32	0.98 (0.13, 7.15)	0.982	0.64 (0.05, 8.68)	0.736
T1	662	0.64 (0.40, 1.04)	0.073	0.72 (0.31, 1.68)	0.453
T2	327	0.95 (0.59, 1.53)	0.831	0.69 (0.37, 1.30)	0.254
T3	72	0.65 (0.27, 1.57)	0.339	0.44 (0.13, 1.47)	0.183
T4	63	0.61 (0.17, 2.16)	0.445	1.43 (0.19, 10.92)	0.728
N stage					
N0	815	0.74 (0.51, 1.07)	0.105	0.84 (0.44, 1.63)	0.615
N1	341	0.74 (0.46, 1.17)	0.198	0.65 (0.38, 1.14)	0.133
M stage					
M0	1083	0.82 (0.59, 1.14)	0.238	0.88 (0.52, 1.48)	0.638
M1	73	0.51 (0.20, 1.27)	0.146	0.32 (0.12, 0.88)	0.027

Abbreviations: CI, confidence interval; HR, hazard ratio; MCC, MCC‐specific survival.

The subgroup analysis results of relationship between gender and clinical prognosis are shown in Table S2. Gender was basically correlated with better OS (75–80 years group: HR = 1.79; 95% CI = 1.25–2.56; *p* = 0.001; 80–85 years group: HR = 1.42; 95% CI = 1.05–1.92; *p* = 0.023; and ≥85 years group: HR = 1.33; 95% CI = 1.03–1.71; *p* = 0.028), and it was significantly associated with MSS in the 75–80 years group (HR = 1.86; 95% CI = 1.14–3.01; *p* = 0.012).

## 4 DISCUSSION

Widely accepted as the best treatment for various solid tumors, surgery is also a preferred option for elderly patients.^22^ As reported in several studies, following a clinical prognosis assessment, surgery is generally effective in elderly patients with solid cancers such as colon cancer,^23^ liver cancer,^24^ pancreatic cancer,^25^ and gastric cancer.^26^ Using subgroup analyses, this study revealed that surgical resection can improve the OS of aged MCC patients and found the potential population that may benefit from surgery.

Previous studies showed that surgery could not improve outcomes and was not significantly correlated with OS and MSS of MCC patients.^20^, ^27^ Yan et al. compared outcomes of patients undergoing different surgical procedures and reported that different surgical types, including LD, EM ≤1 cm, 1 cm < EM ≤ 2 cm, and EM >2 cm, were not linked with differences in patient prognosis.^19^ In the present real‐world clinical study, we observed that surgical resection was associated with improved OS, which is consistent with the results of previous studies regarding the effects of surgery on the outcomes of elderly patients with oral tongue squamous cell carcinoma^21^ or pancreatic cancer.^25^ We found that surgical management was associated with a favorable prognosis and could improve OS in the ≥85 years group, which may be attributed to the following factors. Older patients suffering from cancer tend to have comorbidities such as lower lung function and cardiovascular diseases.^28^ For MCC patients over 85 years old, surgeons are likely to make a thorough analysis of the patients’ possible complications and the functions of their vital organs before surgical treatment. And then, positive measures are taken to prevent and control these complications. For example, close serum electrolyte level monitoring and correction of fluid and electrolyte imbalances are normally conducted to avoid cardiovascular complications.^29^ Smoking cessation and breathing exercises, which are beneficial for maintaining normal lung function, are proposed.^29^ Among older MCC patients, surgery improves the OS rate but not disease‐specific survival rates, which could be attributed to the effective management of comorbidities, and/or an appropriate preoperative selection of patients. Our hypothesis is supported by the following phenomena: the results after PSM showed that under comparable preoperative conditions, surgery exerted no significant effect on the prognosis of elderly patients. However, surgery may be of positive significance for elderly patients, especially patients with advanced MCC older than 85 years old. Even partial tumor resection can reduce the impact of tumor load on the whole body, which is of great significance to prolong the patient's OS rate. Other unidentified factors may also play a role. Besides, our study further supports that the female patients with MCC have a survival advantage over males, which may be related to the inherent immune differences between males and females.^30^

Comprehensive screening of elderly patients with MCC should be conducted to select patients with a high tolerance for surgical trauma and better general physical condition that can benefit from surgical strategies. Accordingly, a comprehensive evaluation of comorbidities, functional status, cognitive status, emotional status, nutritional status, and social support should be made in elderly patients by some tools, such as the Charlson Comorbidity Index, before the decision‐making in surgical treatment.^31^, ^32^ Elderly individuals are more susceptible to the side effects of cancer therapy, which is associated with an increased incidence of comorbidities, decreased body functions, and the development of neuropsychological problems.^33^ It is unclear whether patients aged 75 years and older with MCC should undergo surgical resection; thus, we should weigh the pros and cons and make medical decisions much more carefully. Surgery is recommended to patients with a favorable prognosis after an effective preoperative evaluation.

The main strength of this study is its relatively large sample size, which allow us to better observe the impact of the surgery on the prognostic outcomes of MCC patients aged 75 years and older. However, the study also has some limitations. First, its retrospective design may have introduced some bias. Second, as other relevant survival data, such as comorbidities, perioperative care, and specific systemic treatment options, are absent from the SEER database, they could not be adjusted in this analysis. Finally, different surgical approaches were not evaluated in this study. All these limitations should be addressed in future studies.

## 5 CONCLUSIONS

Our results suggest that age and surgery were independent prognostic factors for MCC patients aged 75 years and older. Surgical resection may enhance the OS of older MCC patients, especially those aged 85 years and older. Nevertheless, this result requires further confirmation in larger prospective clinical trials.

## CONFLICT OF INTEREST

The authors declare no relevant conflict of interest.

## AUTHOR CONTRIBUTIONS

(1) Conception and design: Bingrong Zhou; (2) Administrative support: Bingrong Zhou; (3) Provision of study materials or patients: Kehui Ren; (4) Collection and assembly of data: Kehui Ren and Xufeng Yin; (5) Data analysis and interpretation: Kehui Ren and Xufeng Yin; (6) Manuscript writing: All authors; and (7) Manuscript revision: All authors.

## ETHICAL APPROVAL STATEMENT

The study was conducted in accordance with the Declaration of Helsinki (as revised in 2013). Since individual information has been removed from all the SEER database, informed consent from patients and approval by the institutional review board were exempted.

## Supporting information

FIGURE S1

FIGURE S2

TABLE S1

TABLE S2

## Data Availability

The data that support the findings of this study are available from the corresponding author upon reasonable request.
