# Supplementary figures and images for: Effects of surgery on survival of patients aged 75 years or older with Merkel cell carcinoma
Source: Cancer Med. 2021 Nov 24;11(1):128–38. doi: 10.1002/cam4.4437 (PMC8704145; doi:10.1002/cam4.4437)

A

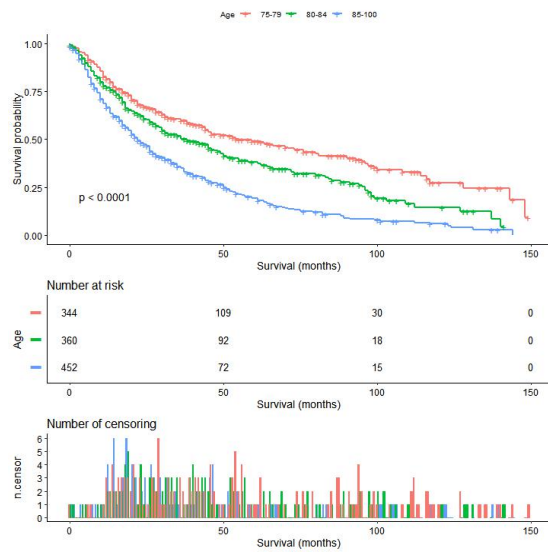

B

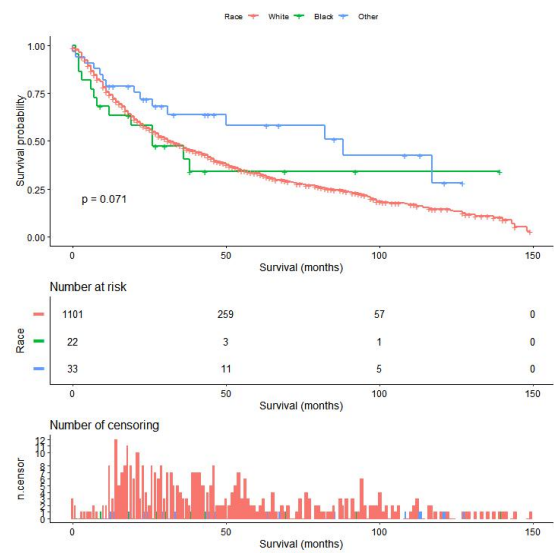

C

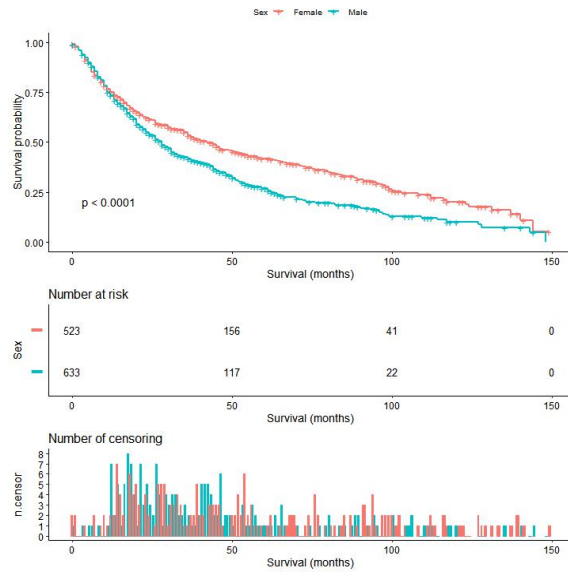

D

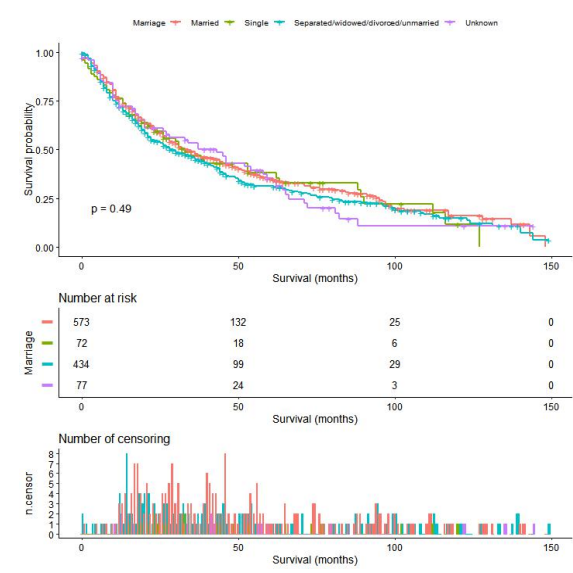

E

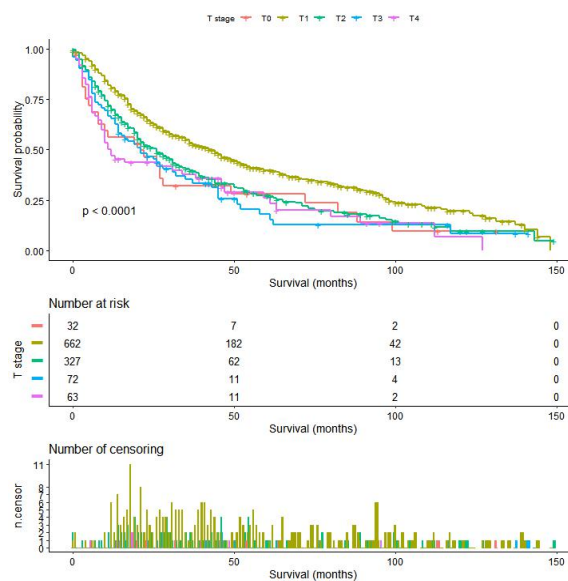

F

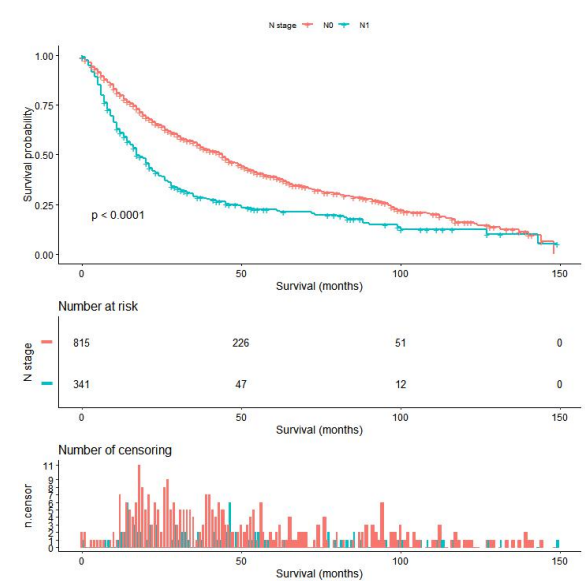

G

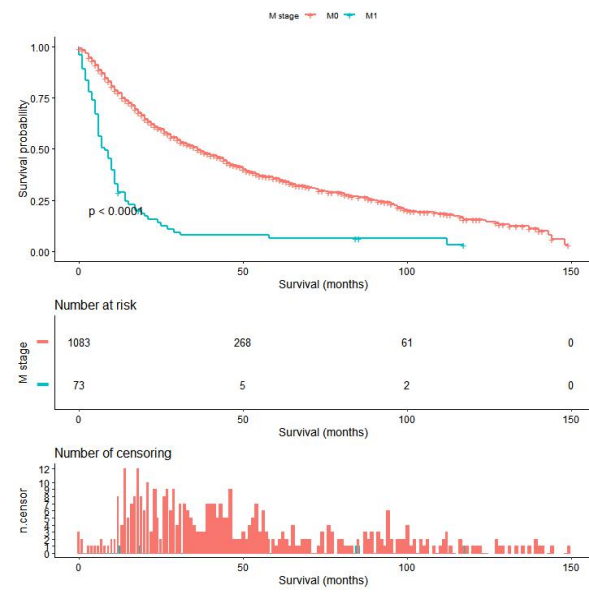

Supplement: Supplementary file 1 — FIGURE S1 [file CAM4-11-128-s002.pdf]

A

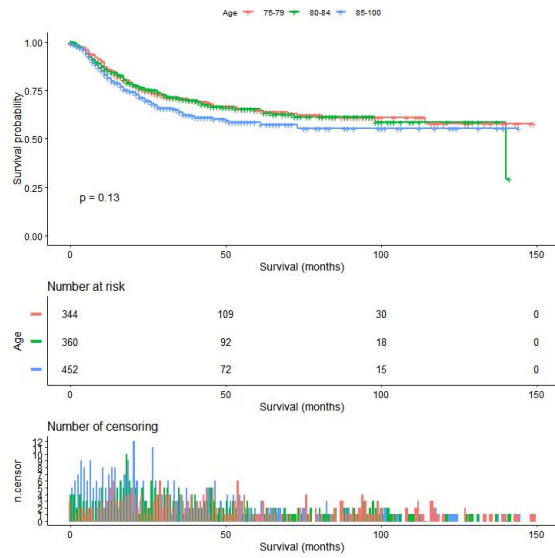

B

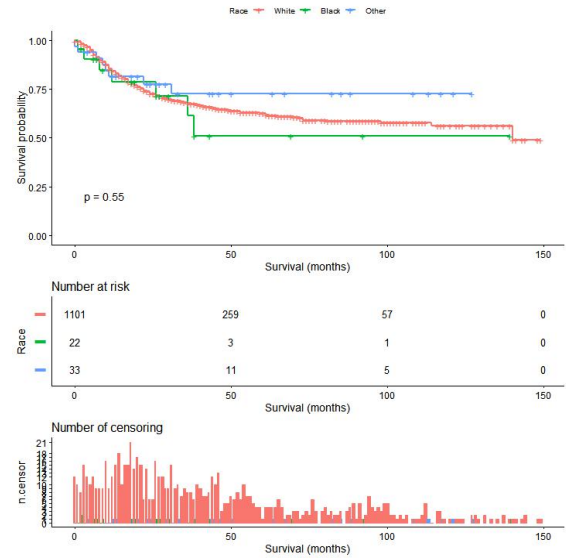

C

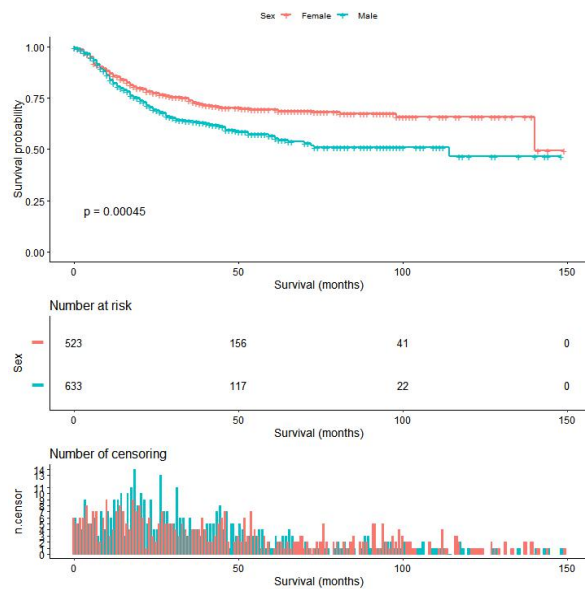

D

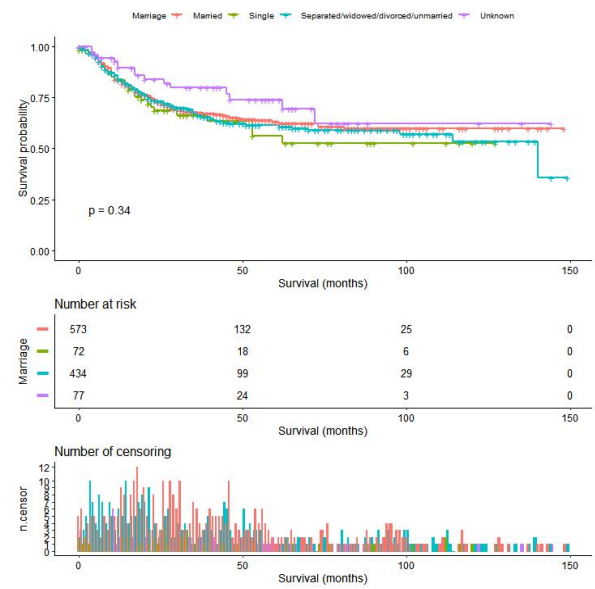

E

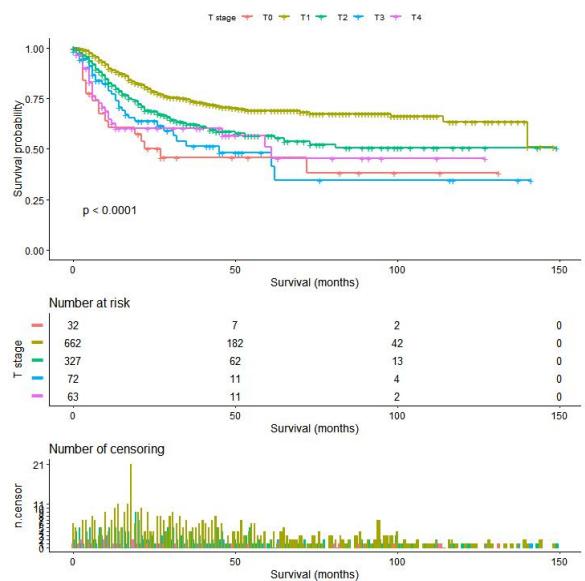

F

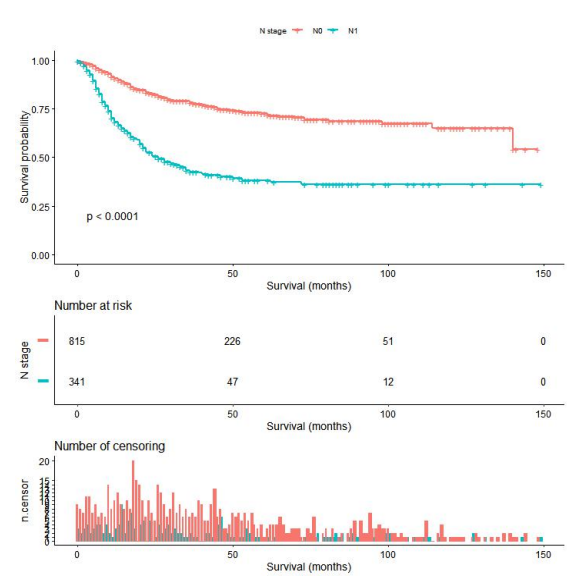

G

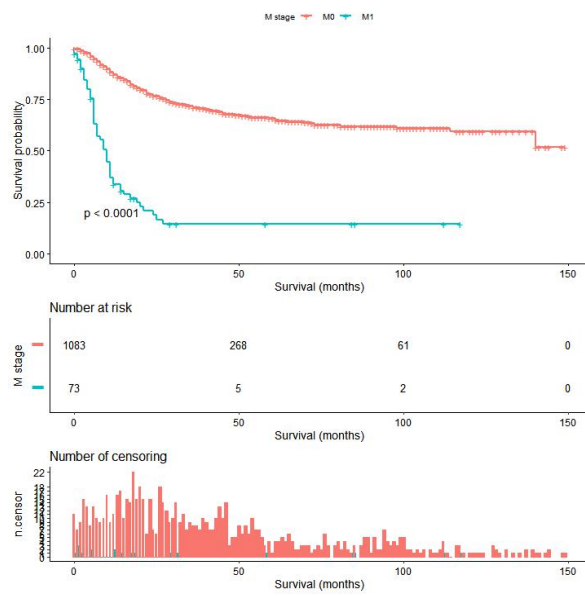

Supplement: Supplementary file 2 — FIGURE S2 [file CAM4-11-128-s003.pdf]
