# Supplementary material for: Effects of surgery on survival of patients aged 75 years or older with Merkel cell carcinoma
Source: Cancer Med. 2021 Nov 24;11(1):128–38. doi: 10.1002/cam4.4437 (PMC8704145; doi:10.1002/cam4.4437)
Supplement: Supplementary file 3 — TABLE S1 [file CAM4-11-128-s001.docx]

**Table S1.** Comparison of basic characteristics of elderly patients with Merkel cell carcinoma between survival and death groups.

| **Overall survival** | **Alive** | **Dead** | ***P*-value** |
| --- | --- | --- | --- |
| **N** | 400 | 756 |  |
| **Survival time (months) [median (inter-quartile range)]** | 40.0 (22.0-65.2) | 17.0 (8.0-36.0) | <0.001 |
| **Age, n (%)** |  |  | <0.001 |
| 75-80 | 165 (41.2%) | 179 (23.7%) |  |
| 80-85 | 135 (33.8%) | 225 (29.8%) |  |
| ≥85 | 100 (25.0%) | 352 (46.6%) |  |
| **Race, n (%)** |  |  | 0.04 |
| White | 373 (93.2%) | 728 (96.3%) |  |
| Black | 9 (2.2%) | 13 (1.7%) |  |
| Other | 18 (4.5%) | 15 (2.0%) |  |
| **Sex, n (%)** |  |  | 0.003 |
| Female | 205 (51.2%) | 318 (42.1%) |  |
| Male | 195 (48.8%) | 438 (57.9%) |  |
| **Marital status, n (%)** |  |  | 0.214 |
| Married | 215 (53.8%) | 358 (47.4%) |  |
| Single | 24 (6.0%) | 48 (6.3%) |  |
| Separated/widowed/divorced/unmarried | 138 (34.5%) | 296 (39.2%) |  |
| Unknown | 23 (5.8%) | 54 (7.1%) |  |
| **Tumor size, n (%)** |  |  | <0.001 |
| <2cm | 252 (63.0%) | 386 (51.1%) |  |
| ≥2cm | 148 (37.0%) | 370 (48.9%) |  |
| **Lymph nodes, n (%)** |  |  | <0.001 |
| No lymph involved | 316 (79.0%) | 499 (66.0%) |  |
| Lymph involved | 84 (21.0%) | 257 (34.0%) |  |
| **Primary site, n (%)** |  |  | 0.002 |
| Face | 158 (39.5%) | 282 (37.3%) |  |
| Head/neck | 30 (7.5%) | 101 (13.4%) |  |
| Trunk | 27 (6.8%) | 67 (8.9%) |  |
| Limbs/shoulder/hip | 179 (44.8%) | 280 (37.0%) |  |
| Other | 6 (1.5%) | 26 (3.4%) |  |
| **T stage, n (%)** |  |  | <0.001 |
| T0 | 6 (1.5%) | 26 (3.4%) |  |
| T1 | 266 (66.5%) | 396 (52.4%) |  |
| T2 | 98 (24.5%) | 229 (30.3%) |  |
| T3 | 16 (4.0%) | 56 (7.4%) |  |
| T4 | 14 (3.5%) | 49 (6.5%) |  |
| **N stage, n (%)** |  |  | <0.001 |
| N0 | 316 (79.0%) | 499 (66.0%) |  |
| N1 | 84 (21.0%) | 257 (34.0%) |  |
| **M stage, n (%)** |  |  | <0.001 |
| M0 | 395 (98.8%) | 688 (91.0%) |  |
| M1 | 5 (1.2%) | 68 (9.0%) |  |
| **AJCC stage, n (%)** |  |  | <0.001 |
| I | 229 (57.2%) | 300 (39.7%) |  |
| II | 80 (20.0%) | 152 (20.1%) |  |
| III | 86 (21.5%) | 236 (31.2%) |  |
| IV | 5 (1.2%) | 68 (9.0%) |  |
| **Surgical procedure, n (%)** |  |  | <0.001 |
| No surgery | 23 (5.8%) | 83 (11.0%) |  |
| LD | 73 (18.2%) | 182 (24.1%) |  |
| EM≤1cm | 140 (35.0%) | 188 (24.9%) |  |
| 1cm＜EM≤2cm | 118 (29.5%) | 234 (31.0%) |  |
| EM＞2cm | 27 (7.8%) | 23 (6.4%) |  |
| Unknow | 18 (5.2%) | 12 (3.3%) |  |
| **Radiation, n (%)** |  |  | 0.872 |
| No/unknown | 202 (50.5%) | 378 (50.0%) |  |
| Yes | 198 (49.5%) | 378 (50.0%) |  |
| **Chemotherapy, n (%)** |  |  | <0.001 |
| No/unknown | 385 (96.2%) | 687 (90.9%) |  |
| Yes | 15 (3.8%) | 69 (9.1%) |  |
| **Distant metastases, n (%)** |  |  | <0.001 |
| No | 395 (98.8%) | 688 (91.0%) |  |
| Yes | 5 (1.2%) | 68 (9.0%) |  |
| **MCC-specific survival** |  |  | <0.001 |
| Alive/dead of other cause | 400 (100.0%) | 414 (54.8%) |  |
| Dead | 0 (0.0%) | 342 (45.2%) |  |
