# Supplementary material for: Effects of surgery on survival of patients aged 75 years or older with Merkel cell carcinoma
Source: Cancer Med. 2021 Nov 24;11(1):128–38. doi: 10.1002/cam4.4437 (PMC8704145; doi:10.1002/cam4.4437)
Supplement: Supplementary file 4 — TABLE S2 [file CAM4-11-128-s004.docx]

**Table S2.** The effect of gender on overall survival and cancer-specific survival based on different subgroup variables.

| **Characteristics** | **N** | **Overall survival** | |  | **MCC-specific Survival** | |
| --- | --- | --- | --- | --- | --- | --- |
|  |  | HR (95% CI) | *P*-value |  | HR (95% CI) | *P*-value |
| **Age** |  |  |  |  |  |  |
| 75-80 years | 344 | 1.79 (1.25, 2.56) | 0.001 |  | 1.86 (1.14, 3.01) | 0.012 |
| 80-85 years | 360 | 1.42 (1.05, 1.92) | 0.023 |  | 1.50 (0.95, 2.37) | 0.082 |
| ≥85 years | 452 | 1.33 (1.03, 1.71) | 0.028 |  | 1.03 (0.69, 1.55) | 0.877 |
| **Primary site** |  |  |  |  |  |  |
| Face | 439 | 1.58 (1.20, 2.07) | 0.001 |  | 1.60 (1.04, 2.48) | 0.034 |
| Head/neck | 132 | 1.62 (0.95, 2.77) | 0.076 |  | 1.73 (0.77, 3.84) | 0.182 |
| Trunk | 94 | 1.24 (0.67, 2.29) | 0.494 |  | 1.85 (0.79, 4.34) | 0.159 |
| Limbs/shoulder/hip | 457 | 1.27 (0.97, 1.65) | 0.077 |  | 1.00 (0.66, 1.54) | 0.987 |
| Other | 34 | 0.70 (0.24, 2.03) | 0.516 |  | 0.43 (0.12, 1.45) | 0.171 |
| **T stage** |  |  |  |  |  |  |
| T0 | 32 | 0.81 (0.28, 2.41) | 0.711 |  | 0.45 (0.13, 1.52) | 0.198 |
| T1 | 662 | 1.54 (1.23, 1.93) | 0.0002 |  | 1.43 (1.00, 2.06) | 0.050 |
| T2 | 327 | 1.09 (0.81, 1.47) | 0.570 |  | 1.19 (0.76, 1.85) | 0.453 |
| T3 | 72 | 2.26 (1.09, 4.70) | 0.029 |  | 1.46 (0.57, 3.74) | 0.428 |
| T4 | 63 | 0.83 (0.36, 1.92) | 0.671 |  | 0.93 (0.25, 3.38) | 0.906 |
| **N stage** |  |  |  |  |  |  |
| N0 | 815 | 1.62 (1.32, 1.98) | <0.0001 |  | 1.68 (1.19, 2.39) | 0.004 |
| N1 | 341 | 1.03 (0.77, 1.37) | 0.864 |  | 1.02 (0.71, 1.46) | 0.912 |
| **M stage** |  |  |  |  |  |  |
| M0 | 1083 | 1.43 (1.21, 1.69) | <0.0001 |  | 1.37 (1.05, 1.78) | 0.019 |
| M1 | 73 | 1.17 (0.56, 2.47) | 0.674 |  | 1.51 (0.67, 3.39) | 0.320 |

CI: confidence interval, HR: hazard ratio.
